# First Crystal Structure of an Aspartame Cocrystal

**DOI:** 10.1021/acs.cgd.5c00373

**Published:** 2025-07-29

**Authors:** Nazanin Fereidouni, Marwah Aljohani, Andrea Erxleben

**Affiliations:** † School of Biological and Chemical Sciences, 8799 University of Galway, Galway H91TK33, Ireland; ‡ Department of Chemistry, College of Science, 48023 Imam Abdulrahman Bin Faisal University, P.O. Box 76971, Dammam 31441, Saudi Arabia; § Synthesis and Solid State Pharmaceutical Centre (SSPC), Limerick V94 T9PX, Ireland

## Abstract

Aspartame crystallizes as very long, thin needles. The crystallization behavior of extreme needle formers not only causes problems in industrial processing and handling but is also of interest in fundamental research. Cocrystallization is a popular approach to expand the solid-state landscape of a compound and often leads to improved physicochemical properties such as stability, dissolution behavior, particle size, and morphology. No crystal structure of an aspartame cocrystal has been reported in the literature up to now. In this work, a comprehensive screening study for aspartame cocrystals was performed. Cocrystals with fumaric acid and 4-hydroxybenzoic acid were detected by powder X-ray diffraction analysis. Growing X-ray suitable cocrystals, however, proved extremely difficult, as both cocrystals, like aspartame, crystallized as very fine needles. Nevertheless, in the case of 4-hydroxybenzoic acid, crystals of sufficient quality for single-crystal X-ray analysis could be grown, and the first crystal structure of an aspartame cocrystal is reported. In the cocrystal aspartame·4-hydroxybenzoic acid dihydrate (**1**), the coformer forms the OH···^–^OOC synthon with aspartame. The aspartame zwitterions in **1** are connected through charge-assisted NH_3_^+^···^–^OOC hydrogen bonds into a spiral along a 2_1_ screw axis, the same structural feature that drives the needle growth of aspartame and that seems to be the reason why the isolation of X-ray-quality cocrystals of aspartame is so challenging.

## Graphical abstract

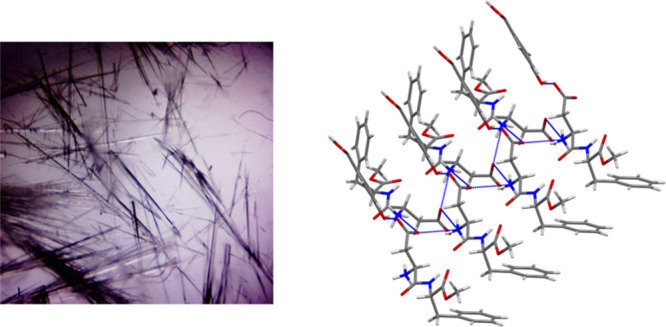

## Introduction

Aspartame, the methyl ester of l-aspartyl-l-phenylalanine, is an FDA-approved artificial, nonsaccharide, inexpensive sweetener that is added to thousands of products.^1^ Four different pseudopolymorphs of aspartame are reported in the CSD;^2^ anhydrous aspartame or form IIB (KETXIR),^3^ aspartame hemihydrate or form IIA (DAWGOX),^4^ and the hydrates IB (ODOBAK)^5^ and IA (EFIFOO, EFIF0001).^5,6^ All forms have a 1D motif of strong charge-assisted hydrogen bonds and crystallize invariably as very fine needles with aspect ratios of up to 10^4^–10^6^. Because of the extreme needle morphologies, form IIA is the only form for which complete single-crystal data could be reported after considerable effort. The structures of the other forms were solved from powder data or have issues with the modeling of disordered lattice water.

Cocrystallization is by now a well-established route to new solid-state forms of pharmaceuticals, agrochemicals, dyes, explosives, and optoelectronic materials with physicochemical properties distinct from those of the parent compounds.^7–10^ In the field of pharmaceutical cocrystals, amino acids and peptides are particularly attractive coformers due to their biocompatibility and safety.^11^ Aspartame has therefore been used as a coformer in a small number of cocrystallization studies with the aim of enhancing the dissolution behavior of a poorly soluble active pharmaceutical ingredient. However, no crystal structure of an aspartame cocrystal has been reported to date. Aspartame has been cocrystallized with glibenclamide^12^ and atorvastatin,^13^ and the structures were predicted using molecular docking, while the product of the cocrystallization of simvastatin and aspartame was characterized by X-ray powder diffraction (XRPD) analysis.^14^ A fourth cocrystal and its XRPD pattern are mentioned in a patent.^15^

The crystal growth of extreme needle formers such as aspartame has been extensively studied, both experimentally and theoretically,^16^ since needle-shaped crystals are notoriously difficult to handle in industrial processing. They have poor flow properties, are difficult to filter and to dry, and break easily, creating fines and affecting product quality and performance.^17^ Therefore, with the aim of gaining a better understanding of the crystallization behavior and solid-state landscape of aspartame, we set out to systematically study the formation of aspartame cocrystals. As the main focus was on fundamental research, we included both GRAS (generally recognized as safe) and non-GRAS coformers in our crystal screen.

## Experimental Section

### Materials

Aspartame, caffeic acid, gallic acid hydrate, paracetamol, zingerone, and 4-hydroxypyridine were purchased from the Tokyo Chemical Industry (TCI Europe). 4-Hydroxybenzoic acid, salicylic acid, l-alanine, 3-aminobenzoic acid, 3-hydroxybenzoic acid, ethyl-4-hydroxybenzoate, 2-hydroxy-1,4-naphthoquinone, 2-hydroxypyridine, vanillic acid, nicotinic acid, maleic acid, D,L-tartaric acid, citric acid, succinic acid, d-alanine, nicotinamide, isonicotinamide, piperazine, 2-aminopyrimidine, and *p*-methoxyaniline were obtained from Sigma-Aldrich. Benzoic acid was obtained from BDH, fumaric acid from Fluka, anthranilic acid from ACROS, dl-leucine from Novabiochem (Merck), and methyl-4-hydroxybenzoate from Alfa Aesar.

All chemicals were used as received without further purification. Analytical-grade methanol and acetone were purchased from Fisher Scientific and used without further purification.

### Infrared (IR) Spectroscopy

FT-IR spectra (650–4000 cm^–1^) were recorded on a PerkinElmer Spectrum 400 fitted with an ATR reflectance attachment and diamond/ZnSe optics. Four coadded scans with a resolution of 4 cm^–1^ were used.

### Differential Scanning Calorimetry

Differential scanning calorimetry (DSC) analysis was performed with a PerkinElmer DSC 4000. The sample was heated in a closed aluminum crucible at a rate of 10 °C/min. The temperature range was 20–300 °C.

### ^1^H NMR Spectroscopy

^1^H NMR spectra were measured with a Joel 400 MHz spectrometer. MestreNova was used for data processing. Chemical shifts in ppm were referenced internally using the residual solvent signals relative to tetramethylsilane (δ = 0 ppm). Coupling constants (*J*) are given in Hertz (Hz).

### X-ray Powder Diffraction

X-ray powder diffraction (XRPD) patterns over the 5–50° (2θ) range were obtained with a Rigaku model Ultima IV diffractometer using Cu–K_α_ radiation (λ = 1.54178 Å; 40 kV, 40 mA).

### Solution Crystallization Experiments

#### General

0.1 mmol of aspartame and 1, 2, or 0.5 mol equiv of the respective coformer were dissolved in 10 mL of solvent (Tables S1 and S2) under stirring at 600 rpm with gentle heating. The solvent was slowly evaporated at room temperature.

#### Crystallization of **1**

Crystals of **1** were prepared using a 1:1 molar ratio of aspartame (29.4 mg, 0.1 mmol) and 4-hydroxybenzoic acid (13.8 mg, 0.1 mmol). The components were dissolved in a methanol/water (1:1) solvent mixture under stirring at 600 rpm with gentle heating. After several days, crystals of **1** formed in the beaker. The crystals were collected by vacuum filtration using a Büchner funnel and Whatman filter paper. The collected crystals were washed with cold methanol to remove residual impurities and dried under a vacuum at room temperature. Yield 32.0%. IR (cm^–1^): IR (cm^–1^): 3313 m (ν­(O–H/N–H)), 1735 s (ν­(C=O)), 1662 s (ν­(C=O), amide), 1548 s, 1447 m, 1383 m, 1361 s, 1259 m, 1225 s (ν­(C–O)), 1158 m, 1124 m, 1080 w, 1030 w, 1004 w, 970 m, 917 m, 817 w, 775 w, 751 w, 698 s. ^1^H NMR (MeOD-*d*_4_): δ 7.87 (d, ^3^ *J* = 8.0, 2H), 7.31–7.22 (m, 5H), 6.81 (m, ^3^ *J*, 2H), 4.69 (dd, *J* = 10.0, 6.0, 1H), 4.01 (dd, *J* = 4.0, 8.0, 1H), 3.70 (s, 3H), 3.21 (dd, *J* = 14.0, 6.0, 1H), 2.98 (dd, *J* = 14.0, 6.0 Hz, 1H), 2.76 (dd, *J* = 18.0, 6.0 Hz, 1H), 2.52 (dd, *J* = 18.0, 10.0 Hz, 1H).

#### Crystallization of (Aspartame)_2_·Fumaric Acid (**2**)

Aspartame (29.4 mg, 0.1 mmol) and fumaric acid (11.6 mg, 0.1 mmol) were dissolved in 10 mL of acetone/water (1:1) with stirring at 600 rpm with gentle heating. After several days, long fibers formed in the beaker that were collected by vacuum filtration using a Büchner funnel and Whatman filter paper, washed with 10 mL of cold water, and dried under vacuum at room temperature. Yield 38.6%. IR (cm^–1^): 3321 m (ν­(O–H/N–H)), 1737 s (ν­(C=O)), 1692 m, 1656 s, 1546 s, 1445 m, 1387 m, 1361 m, 1261 s, 1217 s (ν­(C–O)), 1148 w, 1122 m, 1098 w, 1076 w, 1034 w, 980 m, 968 m, 925 m, 875 w, 821 w, 696 m, 632 s. ^1^H NMR: (MeOD-*d*_4_) δ 7.29–7.18 (m, 5H), 6.71 (s, 1H), 4.67 (dd, *J* = 10.0, 6.0 Hz, 2H), 4.01 (dd, *J* = 4.0, 8.0, 2H), 3.68 (s, 1H), 3.21 (dd, *J* = 14.0, 6.0, 2H), 2.96 (dd, *J* = 14.0, 6.0, 2H), 2.78 (dd, *J* = 18.0, 6.0, 2H), 2.56 (dd, *J* = 18.0, 10.0, 2H).

### Stability of 1

The isolated crystals were stored in a desiccator at ambient temperature (20 ± 2 °C). After 2 weeks, the crystals were analyzed by X-ray powder diffraction. A saturated solution of K_2_CO_3_ was used to maintain a relative humidity of 56%.

### Crystallography

Single-crystal data of **1** were collected at room temperature on an Oxford Diffraction Xcalibur system (Oxfordshire, UK) using graphite-monochromated Mo-K_α_ radiation (λ = 0.71073 Å). The structure was solved by intrinsic phasing and refined by full-matrix least-squares on *F* ^2^ using SHELXT^18^ and SHELXL 2018/3 ^19^ within the Olex2 program suite.^20^ Non-hydrogen atoms were refined anisotropically. The hydrogen atoms were placed in geometrically determined positions and refined as riding atoms with isotropic displacement factors equivalent to 1.2 times those of the atom to which they were attached. The disordered aromatic ring carbons and carboxyl oxygens were modeled with fixed 0.65 and 0.35 site occupancies. The structural drawings were made with Mercury.^21^

## Results and Discussion

A comprehensive screen for aspartame cocrystals was performed by the slow evaporation of methanol, methanol/water, and acetone/water solutions containing equimolar amounts of aspartame and the respective coformer. Initially, 21 coformers were tested, including aromatic carboxylic acids (benzoic, caffeic, gallic, salicylic, 4-hydroxybenzoic, anthralinic, 3-aminobenzoic, and nicotinic acid), poly­(carboxylic acid)­s (maleic, fumaric, D,L-tartaric, succinic, and citric acid), heterocyclic bases (nicotinamide, isonicotinamide, 2-aminopyrimidine), and amines (piperazine, *p*-methoxyaniline) as well as α-amino acids (l-alanine, d-alanine, and d,l-leucine). The details of all cocrystallization experiments and results are summarized in Table S1. Despite all efforts, only the use of 4-hydroxybenzoic acid and fumaric acid as coformers was successful. Fine needles of aspartame·4-hydroxybenzoic acid dihydrate (**1**) could be isolated from an aqueous methanol solution, and the crystal structure could be obtained. Crystal data and refinement details are listed in Table 1. Evidence for cocrystal formation between aspartame and fumaric acid is the XRPD pattern (Figure S1) and the IR spectrum (Figure S2) of the isolated cocrystallization product, which showed clear differences from the starting compounds. The ^1^H NMR spectrum (Figure S3) showed aspartame and fumaric acid peaks, and the integrals indicated a 2:1 (aspartame:fumaric acid) composition of the cocrystal. Only very thin needles of (aspartame)_2_·fumaric acid (**2**) could be grown that were not diffracting. All other crystallization experiments gave powders or crystals of the individual coformer or aspartame hydrate, and XRPD, IR, and/or ^1^H NMR analyses did not reveal any other cocrystal.

**Table 1:** Crystal Data of **1**

	**1**
formula	C_21_H_28_N_2_O_10_·C_7_H_6_O_3_·2H_2_O
*M*_r_	468.45
crystal color and habit	colorless needle
crystal size (mm)	0.68 × 0.08 × 0.02
crystal system	monoclinic
space group	*P*2_1_
*a* [Å]	15.4735(8)
*b* [Å]	4.8533(2)
*c* [Å]	16.0760(8)
β [°]	102.012(5)
*V* [Å^3^]	1180.83(10)
*Z*	2
*D*_calc_ (g cm^–3^)	1.318
temperature (K)	293.0(2)
no. measd. reflections	10134
no. unique refl. (*R*_int_)	4904 (0.0378)
no. obs. reflections	3130
final *R*_1_, w*R*_2_ (obs. refl.)	0.0680, 0.1504
goodness-of-fit (obs. refl.)	1.020

The asymmetric unit of **1** contains one aspartame molecule, one 4-hydroxybenzoic acid molecule, and two water molecules of crystallization (Figure 2a). The terminal amino group of aspartame is protonated while the carboxyl group of the aspartate side chain is deprotonated, so that aspartame is in the expected zwitterionic form as observed for all aspartame pseudopolymorphs. The deprotonation of the carboxyl group is evident from the C–O bond distances of 1.224(6) and 1.264(6) Å, which are between the typical values of C–O single and C=O double bonds. The aspartame dipeptide is in the typical trans conformation with ω = 176.5(4)° (Figure 1). The phenylalanine residue adopts a +*gauche* conformation [χ_1_ = 63.1(5)°] while the aspartate residue has a −*gauche* conformation [χ_1_ = −69.0(5)°]. The ψ and φ angles of the dipeptide are 152.2(4)° and −153.5(4)°, respectively. Overall, the conformation of aspartame in the cocrystal is very similar to that of aspartame IIA^4^, IIB^3^, IA^6^, and IB^5^ (Table 2).

**Figure 1:**
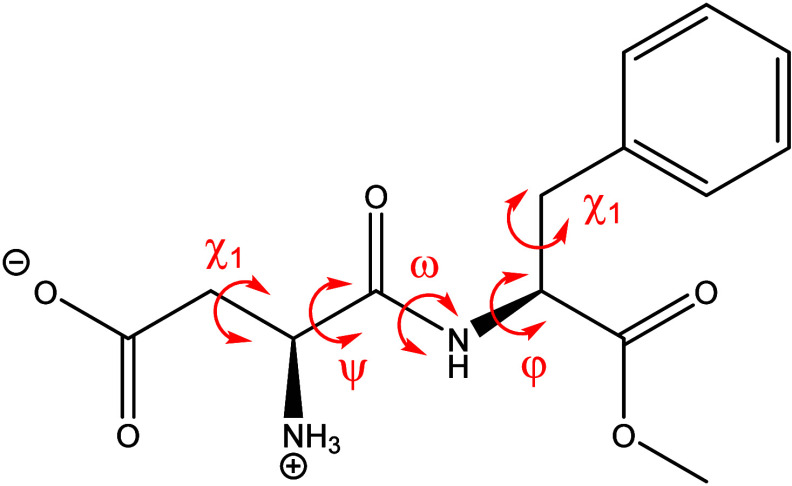
Molecular structure of aspartame with torsional angles.

**Table 2:** Torsional Angles (**°**) of Aspartame in **1** and in the Aspartame Pseudo-Polymorphs

**torsional angle**	**cocrystal**	**IIA (DAWGOX)** ^4^	**IB (ODOBAK)** ^5^	**IA (EFIFOO01)** ^6^	**IIB (KETXIR)** ^3^
ω	176.5	–178.5	–177.4; 175.7; 171.6^a^	173.8; 176.5; −179.9^a^	175.9; 179.2^b^
ψ	152.2	148.9	157.4; 163.3; 152.0^a^	154.9; 151.3; 152.6^a^	139.6; 170.0^b^
φ	–153.5	–156.8	–158.6; −150.2; −148.0^a^	–159.8; −152.0; −158.6^a^	–151.6; −159.8^b^
Asp-χ_1_	–69.0	–71.7	–64.7; −59.8; −78.4^a^	–71.0; −65.9; −67.3^a^	–81.0; −66.6^b^
Phe-χ_1_	63.1	61.7	62.2; 60.2; 71.5^a^	53.3; −56.5; 61.5^a^	71.3; 62.6^b^

a Three crystallographically independent molecules.

b Two crystallographically independent molecules.

The packing of the molecules in the crystal lattice and the hydrogen bonding pattern are shown in Figure 2b,c. Aspartame and 4-hydroxybenzoic acid interact with each other through the OH···^–^OOC synthon between the phenol group of 4-hydroxybenzoic acid and the aspartate side chain. The carboxyl group of 4-hydroxybenzoic acid is disordered over two positions with site occupancies of 0.65 and 0.35 and participates in hydrogen bonding with a lattice water molecule. The C–O bond lengths of 1.367(12)/1.379(18) and 1.191(12)/1.201(18) Å are consistent with a protonated carboxyl group. The ammonium group of aspartame forms three hydrogen bonds: one with water of crystallization and two with the carboxylate groups of adjacent aspartame molecules. The carboxylate oxygen of aspartame that does not interact with the ammonium groups of the neighboring aspartame molecules is involved in hydrogen bonding with the phenol group of the coformer and with both water molecules of crystallization. In contrast to aspartame hydrate and hemihydrate, the water molecules are not disordered. One water molecule forms hydrogen bonds with the NH_3_^+^ group of aspartame and the carboxyl group of 4-hydroxybenzoic acid, while the other one bridges the COOH and COO^–^ groups of aspartame and 4-hydroxybenzoic acid.

**Figure 2:**
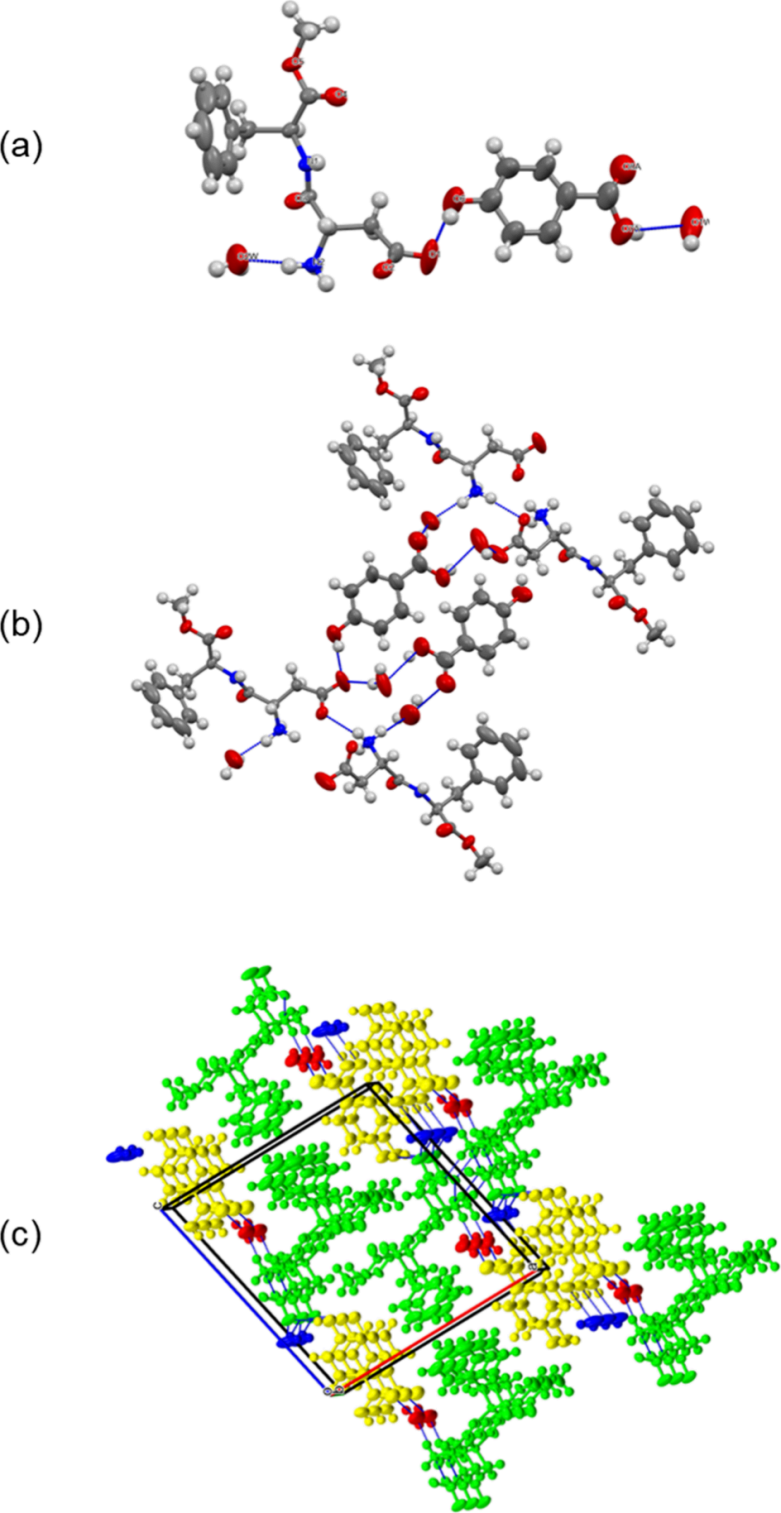
(a) Asymmetric unit of **1**. Ellipsoids are drawn at the 50% probability level. (b) Hydrogen bonding and (b) packing diagram of **1**. Green: aspartame; yellow: 4-hydroxybenzoic acid; red and blue: water of crystallization. For the sake of clarity, only the major components of the disordered phenyl ring and carboxyl group of 4-hydroxybenzoic acid are shown.

The hydrogen-bonded aspartame zwitterions are arranged in a spiral along the 2_1_ screw axis in the *b* direction. While all aspartame pseudopolymorphs contain chains of aspartame zwitterions with strong charge-assisted NH_3_^+^···^–^OOC hydrogen bonds, there are important differences between the arrangement of the zwitterions in the cocrystal and the four aspartame structures^4–6^ (Figure 3). In **1**, the zwitterions are packed as double chains (Figure 3a). Intra- and interchain NH_3_^+^···^–^OOC hydrogen bonds of 2.794(5) and 2.844(4) Å length generate a 1D motif of fused ***R***_**3**_^**2**^(**10**) rings in the *b* direction. The closest resemblance is aspartame hemihydrate (DAWGOX), in which the zwitterions also form double chains. However, in contrast to **1**, both carboxylate oxygens participate in hydrogen bonding with neighboring NH_3_^+^ groups, and fused ***R***_**6**_^**3**^(**12**) rings extend in the direction of the shortest axis (Figure 3b). The two crystallographically independent zwitterions in anhydrous aspartame (KETXIR) pack in single chains (Figure 3c). Aspartame hydrate 1B (ODOBAK) has three crystallographically indendent zwitterions A, B, C that are connected into infinite A···B···C, A···A, and B···B chains (Figure 3d), while in aspartame hydrate 1A (EFIFOO01) water molecules are part of a double chain with aspartame zwitterions and water molecules forming ***R***_**16**_^**16**^(**44**) rings (Figure 3e). Despite these differences in the arrangement of the zwitterions, the strong charge-assisted NH_3_^+^···^–^OOC hydrogen bonds give rise to highly anisotropic growth in all cases, and like aspartame, **1** crystallizes as very fine needles with extended growth along the shortest axis, as shown in Figure 4a.

**Figure 3:**
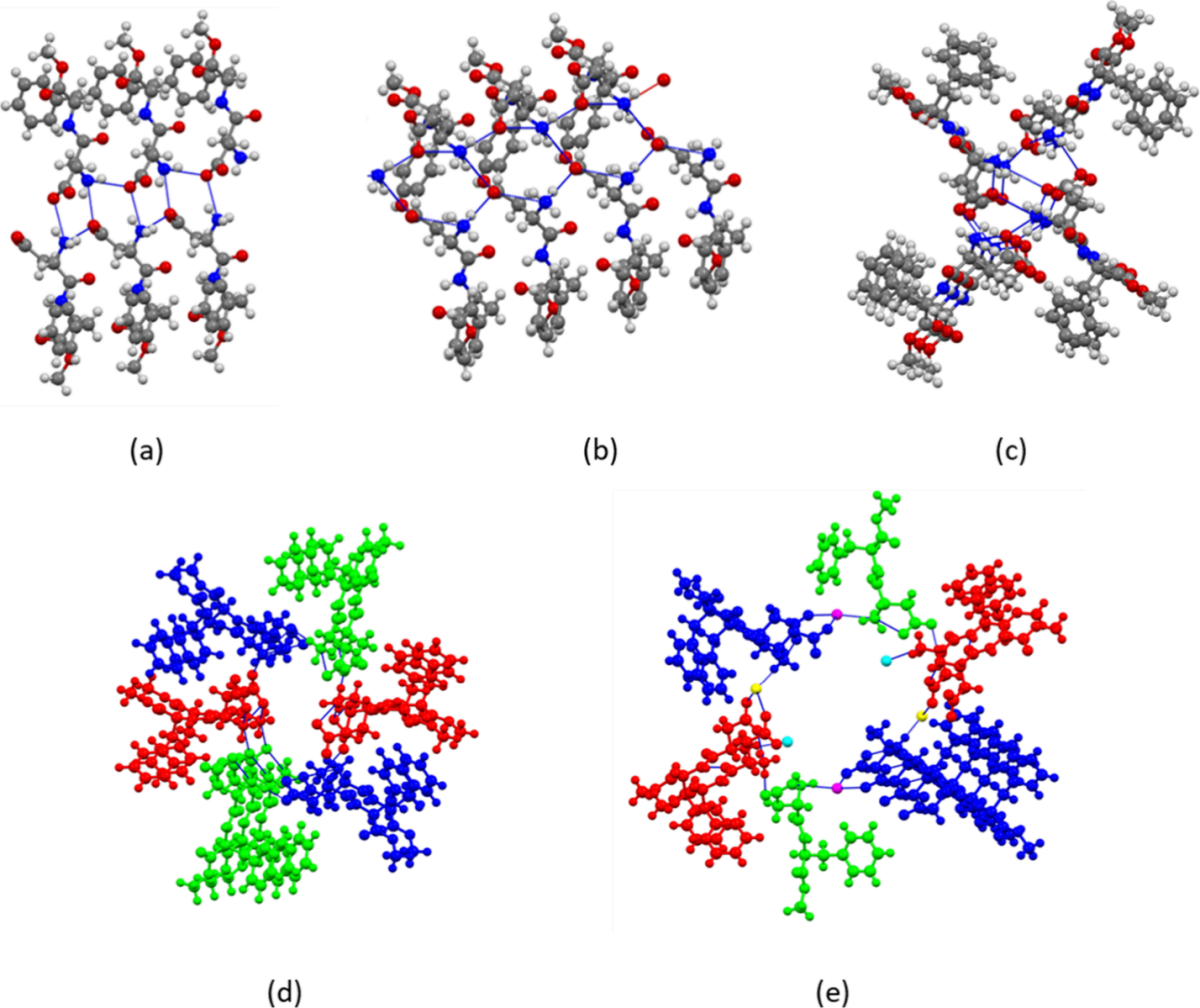
Arrangement of aspartame zwitterions in the solid-state structures of (a) **1**, (b) aspartame hemihydrate (DAWGOX)^4^, (c) anhydrous aspartame (KETXIR)^3^, (d) aspartame hydrate IB (ODOBAK)^5^ and (e) aspartame hydrate IA (EFIFOO01)^6^. In (e), water molecules of crystallization are part of the zwitterion chain. Colors in (d) and (e) are by symmetry equivalence.

**Figure 4:**
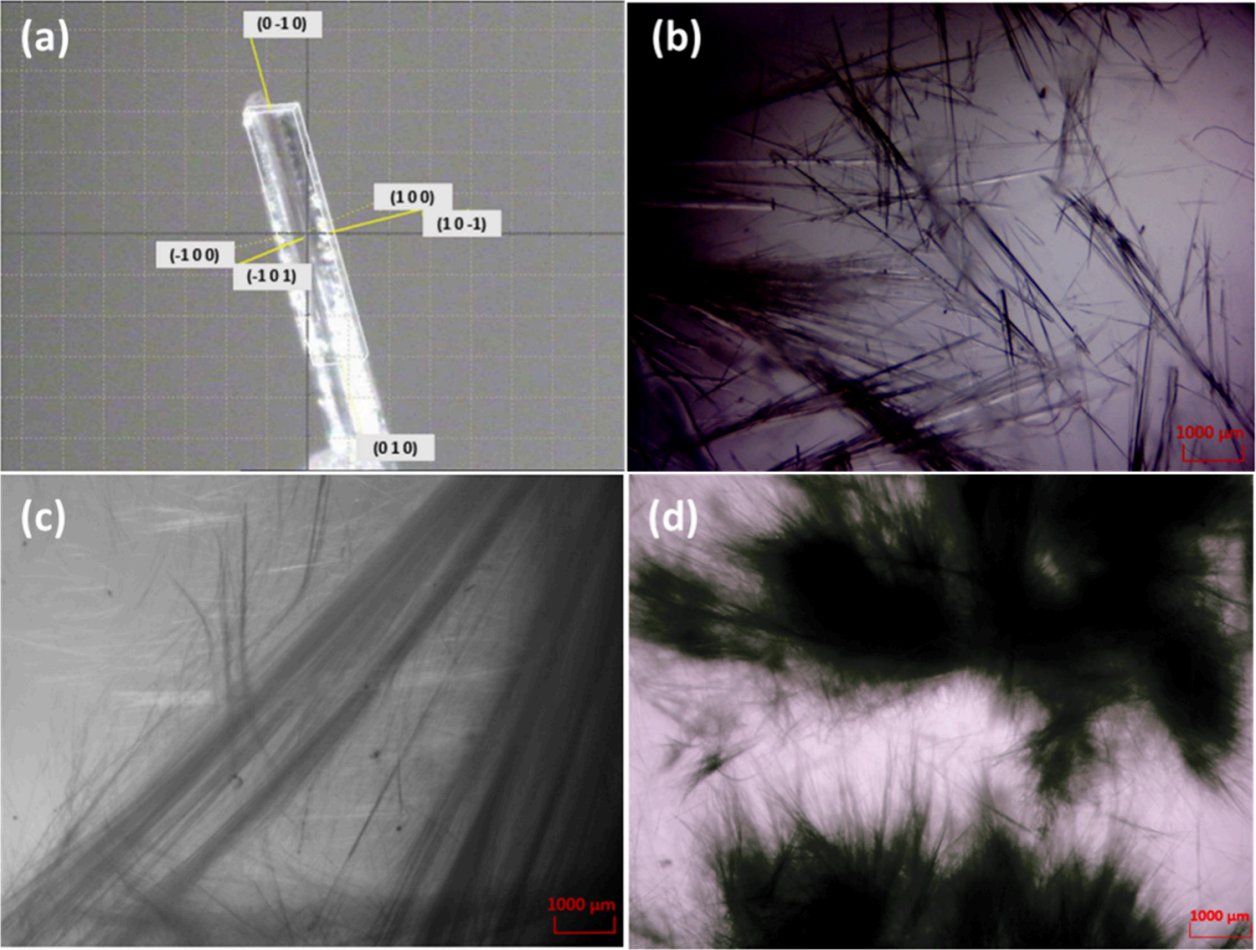
(a) Face-indexing of a crystal of **1** mounted on the diffractometer. (b) **1**, (c) **2**, and (d) aspartame crystallized from aqueous methanol solution.

The extreme needle growth of aspartame was studied by Meekes and co-workers.^6,22^ Kinetic Monte Carlo simulations indicated a rough growth mechanism for the top faces, while large nucleation barriers were found for the side faces of aspartame IA crystals. In form IIA, four zwitterionic chains assemble into an infinite column with a hydrophilic core (NH_3_^+^, COO^–^) and a hydrophobic exterior (phenyl), while form IB contains stacked trimer units that assemble around a pseudo 6_1_ screw axis. These columnar structures can explain the tendency of aspartame to crystallize as ultrathin needles.^3^ Civati et al. quantified the interactions within the columns of form IIA using Pixel calculations and suggested that the extreme anisotropic growth is promoted by the high percentage of atoms in van der Waals contact with their chain neighbors and the high interaction energy of −136.3 kJ mol^–1^ due to the strong charge-assisted hydrogen bonding between the zwitterions.^23^ We cannot perform Pixel calculations for **1**, as the asymmetric unit contains more than two molecules. However, it is reasonable to assume a similarly strong interaction between the zwitterions of the 2_1_ spiral in **1** as in aspartame IIA. Pictures of crystalline samples of aspartame, **1** and **2**, are shown in Figure 4b–d. It is clear that cocrystallization did not change the needle morphology, although **1** forms needles thicker than those of **2** and aspartame. The absence of a change in morphology is a consequence of the fact that the main synthon between the aspartame zwitterions is retained in compound **1**.

To assess the purity of the cocrystals and to determine whether the X-ray structure is representative of the bulk sample, the bulk sample was isolated by filtration and characterized by ^1^H NMR spectroscopy and XRPD. The integration of the ^1^H NMR signals (Figure S4) confirmed the 1:1 composition. The XRPD pattern of the isolated crystals was a good match to the theoretical pattern calculated from the single crystal data (Figure S5). Differential scanning calorimetry analysis of the cocrystal showed six endotherms and one exotherm in the 125–250 °C range (Figure S6) that were not further assigned but show that the aspartame in **1** decomposes on heating, similar to what is described in the literature for aspartame: At higher temperatures, aspartame has been reported to decompose to aspartylphenylalanine or its ketopiperazine and then to aspartic acid and phenylalanine.^3,4^ The stability of the cocrystal at 56% relative humidity and room temperature was monitored over 2 weeks. No change in the XRPD pattern was observed (Figure S7).

As the crystal structure of **1** featured the phenol-carboxylate synthon, we performed a second round of cocrystallization experiments, this time focusing on coformers containing a phenol group or an OH group attached to a pyridine ring, including 3-hydroxybenzoic acid, ethyl-4-hydroxybenzoate, paracetamol, zingerone, 2-hydroxy-1,4-naphthoquinone, 2-hydroxypyridine, 4-hydroxypyridine, methyl-4-hydroxybenzoate, and vanillic acid (Table S2). None of the solution crystallizations yielded another cocrystal. In all cases, crystals of either aspartame or the coformer were obtained.

## Conclusions

In conclusion, we have reported the first structurally characterized cocrystal of aspartame as a result of a comprehensive screening study. The strong charge-assisted hydrogen bonding between the aspartame zwitterions that drives the extreme anisotropic growth of aspartame is retained in the cocrystal structure, which may explain why the isolation of X-ray suitable single cocrystals of aspartame is so challenging. Apparently, out of the wide range of coformers tested, none were able to compete with the primary synthon between aspartame zwitterions. It seems that using 4-hydroxybenzoic acid as the coformer was successful because the coformer columns fit with the aspartame chains in the crystal packing. However, because the coformer did not disrupt the aspartame interactions, it did not significantly improve the morphology. Cocrystallization does not appear to be a promising approach to break the needle growth of aspartame.

## Supplementary Material



## Notes

Details of the cocrystal screen, XRPD patterns, ^1^H NMR and IR spectra of **1** and (aspartame)_2_·fumaric acid, and DSC plot of **1** (PDF)
