# Supplementary material for: First Crystal Structure of an Aspartame Cocrystal
Source: Cryst Growth Des. 2025 Jul 29;25(15):5954–9. doi: 10.1021/acs.cgd.5c00373 (PMC12333019; doi:10.1021/acs.cgd.5c00373)
Supplement: Supplementary file 1 [file cg5c00373_si_001.pdf]

First Crystal Structure of an Aspartame Cocrystal

Nazanin Fereidouni,[1] Marwah Aljohani,[2] and Andrea Erxleben[*1,3]

[1] School of Biological and Chemical Sciences, University of Galway, Galway, H91TK33, Ireland

[2] Department of Chemistry, College of Science, Imam Abdulrahman Bin Faisal University, P.O. Box 76971, Dammam 31441, Saudi Arabia

[3] Synthesis and Solid State Pharmaceutical Centre (SSPC), Limerick, V94 T9PX, Ireland

*Corresponding author email address: andrea.erxleben@nuigalway.ie

Supporting Information

Table S1. Details of the cocrystal screening study.

Coformer	Aspartame : Coformer Ratio	Solvent	Result
benzoic acid	1:1	methanol	crystallization of benzoic acid (BNZAC)
	2:1	methanol	thin needles, not diffracting
	1:1	acetone	very thin fibers
	1:1	methanol/water (1:1)	crystallization of benzoic acid (BNZAC)
	1:1	acetone/water (1:1)	crystallization of benzoic acid (BNZAC)
caffeic acid	1:1	methanol	crystallization of caffeic acid (FENSOG)
	2:1	methanol	fibers
	1:1	acetone	powder
	1:1	methanol/water (1:1)	crystallization of caffeic acid (FENSOG)
	1:1	acetone/water (1:1)	crystallization of caffeic acid (FENSOG)
gallic acid hydrate	1:1	methanol	powder
	1:1	acetone	thin needles, not diffracting
	1:1	methanol/water (1:1)	powder
	1:1	acetone/water (1:1)	oil
salicylic acid	1:1	methanol	powder
	1:1	acetone	powder
	1:1	methanol/water (1:1)	fibers
	1:1	acetone/water (1:1)	powder
4-hydroxybenzoic acid	1:1	methanol	powder
	1:1	acetone	powder
	1:1	methanol/water (1:1)	cocrystal
	1:1	acetone/water (1:1)	cocrystal
nicotinic acid	1:1	methanol	powder
	1:1	acetone	thin needles, not diffracting
	1:1	methanol/water (1:1)	fibers
maleic acid	1:1	methanol	sticky powder
	1:1	acetone	oil
	1:1	methanol/water (1:1)	oil
	1:1	acetone/water (1:1)	oil
D,L-tartaric acid	1:1	methanol	powder
	1:1	acetone	oil
	1:1	methanol/water (1:1)	oil

	1:1	acetone/water (1:1)	oil
fumaric acid	1:1	methanol	powder
	1:1	acetone	powder
	1:1	acetone/water (1:1)	thin needles, not diffracting
citric acid	1:1	methanol	powder
	1:1	acetone	oil
	1:1	methanol/water (1:1)	fibers
	1:1	acetone/water (1:1)	oil
succinic acid	1:1	methanol	powder
	1:1	acetone	powder
	1:1	methanol/water (1:1)	powder
	1:1	acetone/water (1:1)	thin needles, not diffracting
anthranilic acid	1:1	methanol	powder
	1:1	acetone	crystallization of aspartame
	1:1	methanol/water (1:1)	powder
	1:1	acetone/water (1:1)	powder
L-alanine	1:1	methanol	crystallization of aspartame hydrate (EFIFOOD)
	1:2	methanol	fibers
	1:1	methanol/water (1:1)	fibers
	1:1	acetone/water (1:1)	powder
D-alanine	1:1	methanol	crystallization of aspartame hydrate (EFIFOOD)
	1:2	methanol	fibers
	1:1	methanol/water (1:1)	powder
	1:1	acetone/water (1:1)	powder
DL-leucine	1:1	methanol	thin needles, not diffracting
	1:1	methanol/water (1:1)	fibers
	1:1	acetone/water (1:1)	powder
3-aminobenzoic acid	1:1	methanol	thin fibers
	1:1	acetone	thin needles, not diffracting
	1:1	methanol/water (1:1)	powder
	1:1	acetone/water (1:1)	powder
nicotinamide	1:1	methanol	thin fibers
	1:1	methanol/water (1:1)	thin fibers
	1:1	acetone	powder

	1:1	acetone/water	thin fibers
isonicotinamide	1:1	methanol	thin fibers
	1:1	methanol/water (1:1)	thin fibers
	1:1	acetone	thin fibers
	1:1	acetone/water	thin fibers
piperazine	1:1	methanol	oil
	1:1	methanol/water (1:1)	oil
	1:1	acetone	oil
	1:1	acetone/water	oil
2-aminopyrimidine	1:1	methanol	thin fibers
	1:1	methanol/water (1:1)	thin fibers
	1:1	acetone	thin fibers
	1:1	acetone/water	thin fibers
p-methoxyaniline	1:1	methanol	thin fibers
	1:1	methanol/water (1:1)	thin fibers
	1:1	acetone	powder
	1:1	acetone/water	oil

Table S2. Details of the screening study with phenol coformers.

Coformer	Aspartame : Coformer Ratio	Solvent	Result
3-hydroxybenzoic acid	1:1	methanol	crystallization of aspartame
ethyl-4-hydroxybenzoate	1:1	methanol	crystallization of aspartame
paracetamol	1:1	methanol	crystallization of aspartame
zingerone	1:1	methanol	crystallization of aspartame
2-hydroxy-1,4-naphthoquinone	1:1	methanol	crystallization of 2-hydroxy-1,4-naphthoquinone
2-hydroxypyridine	1:1	methanol	crystallization of aspartame
4-hydroxypyridine	1:1	methanol	crystallization of aspartame
methyl-4-hydroxybenzoate	1:1	methanol	crystallization of aspartame
vanillic acid	1:1	methanol	crystallization of aspartame

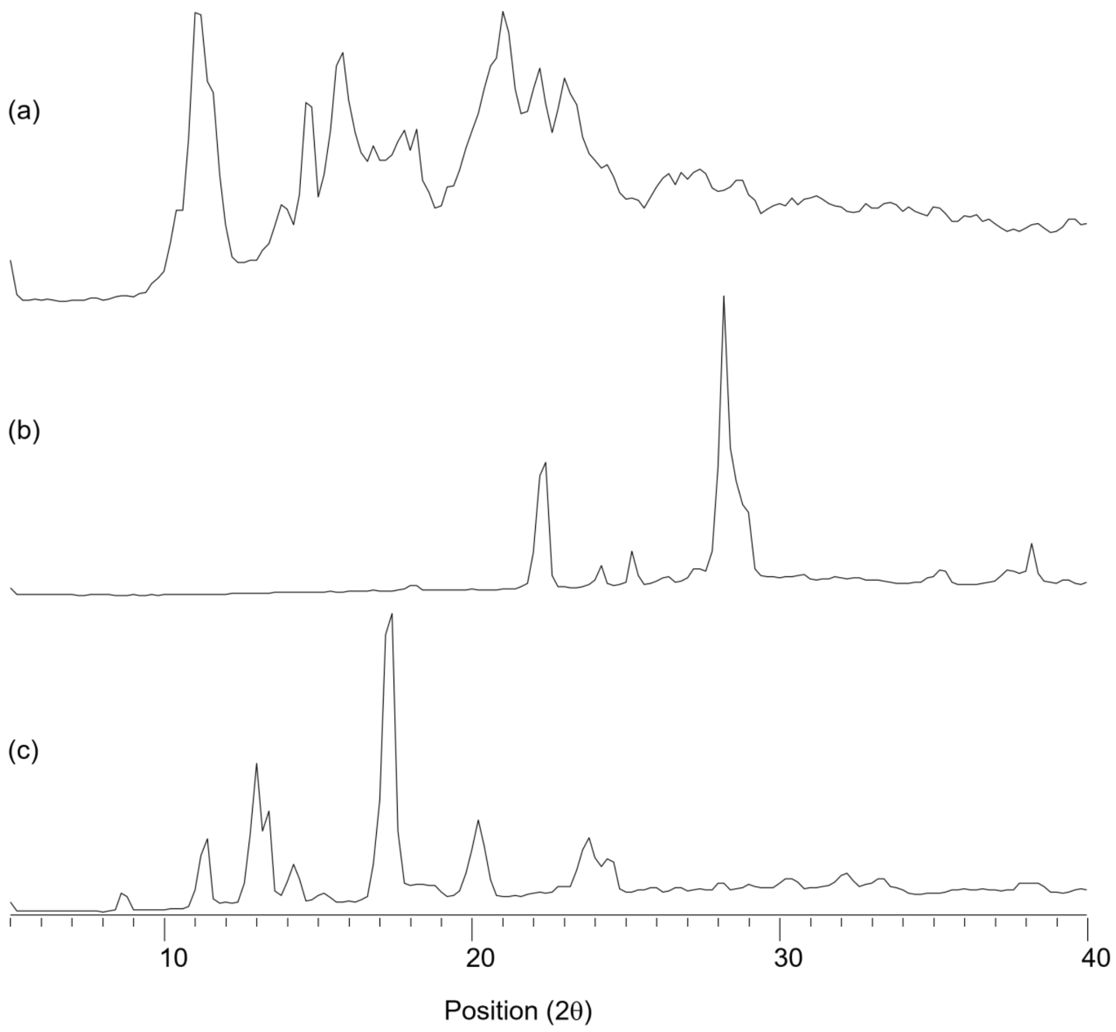

Figure S1. XRPD patterns of (a) aspartame, (b) fumaric acid and (c) the isolated cocrystallization product of aspartame and fumaric acid.

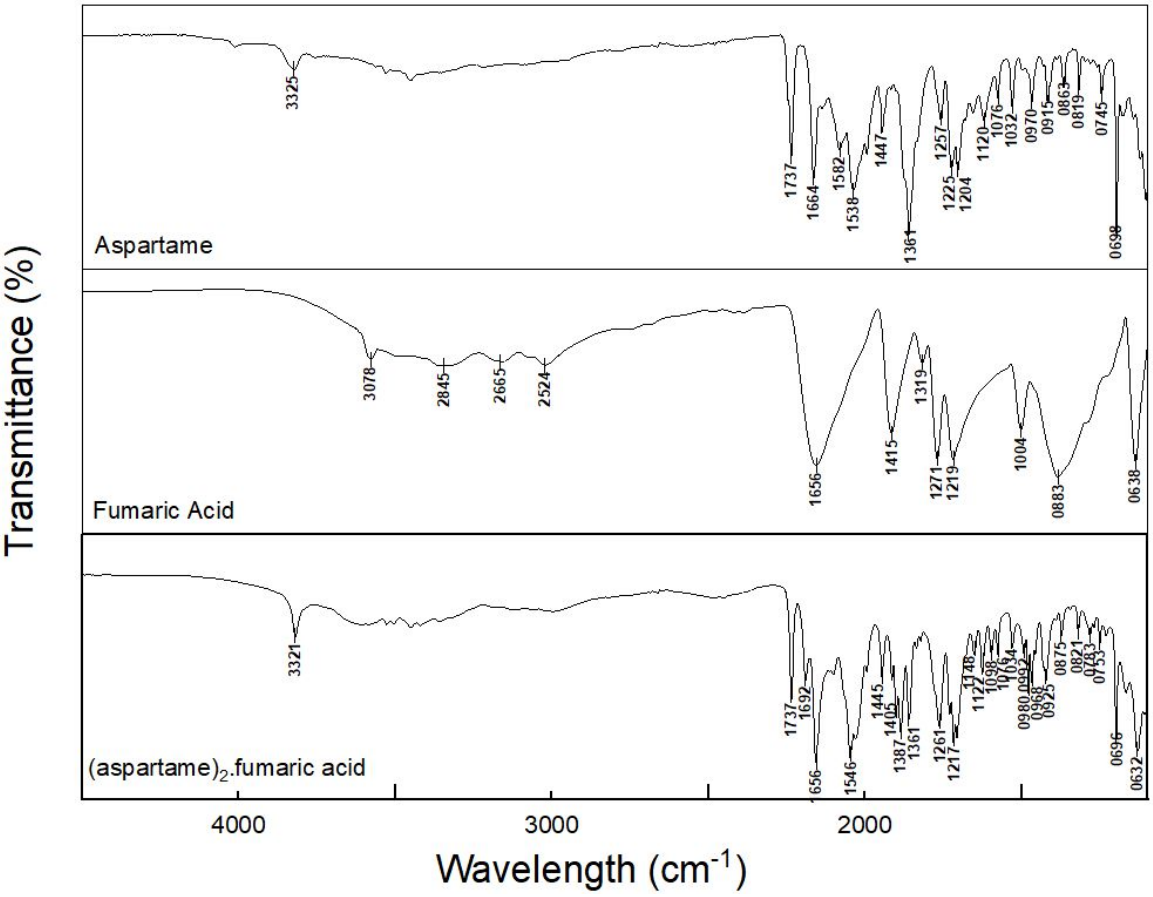

Figure S2. IR spectra of aspartame, fumaric acid and the isolated cocrystallization product of aspartame and fumaric acid.

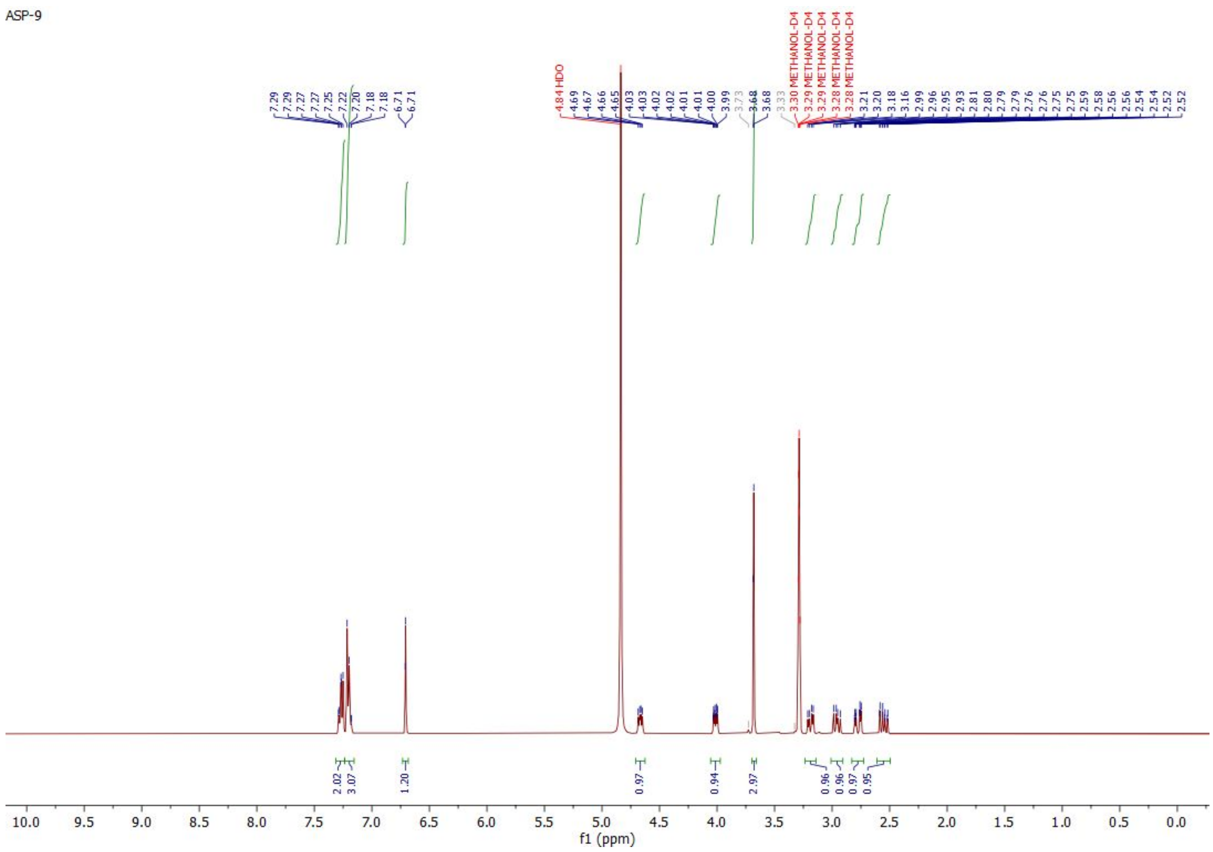

Figure S3. ^{1}H NMR spectrum of the isolated cocrystallization product of aspartame and fumaric acid in MeOD.

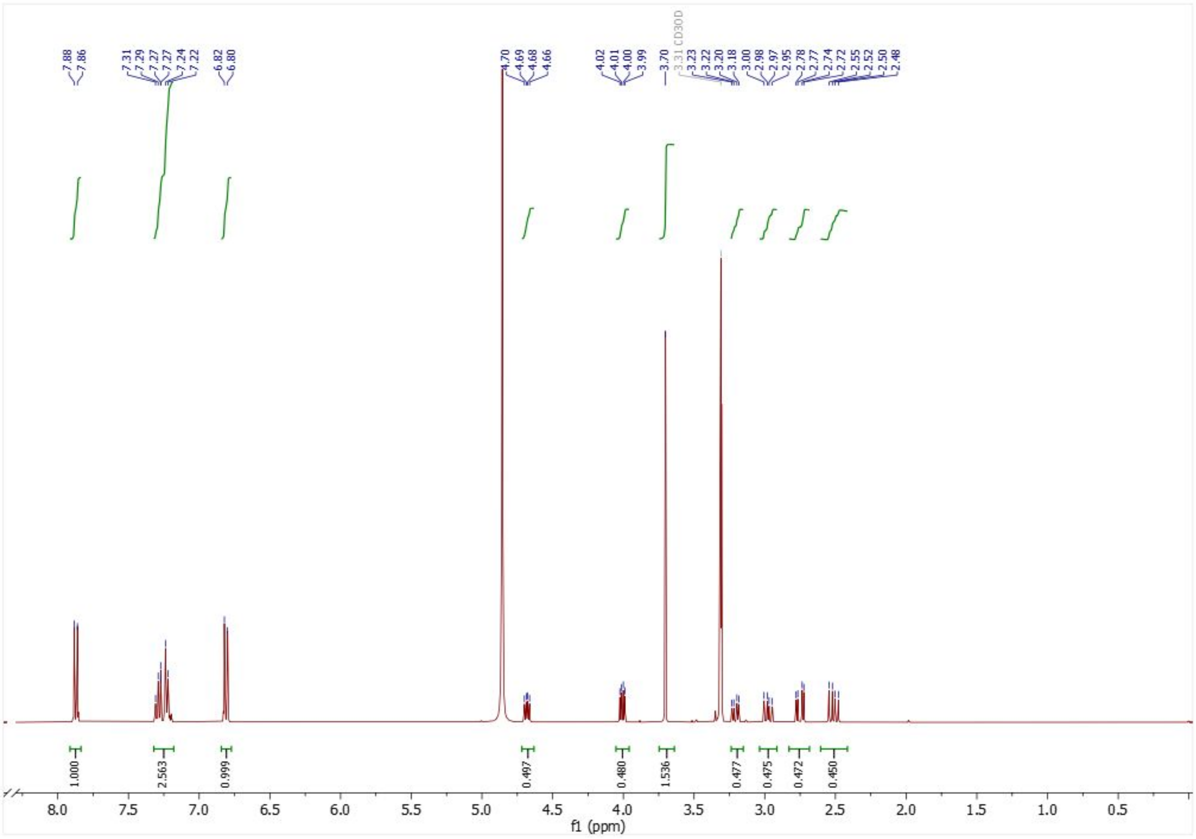

Figure S4. ^1H NMR spectrum of the isolated crystals of **1** in MeOD.

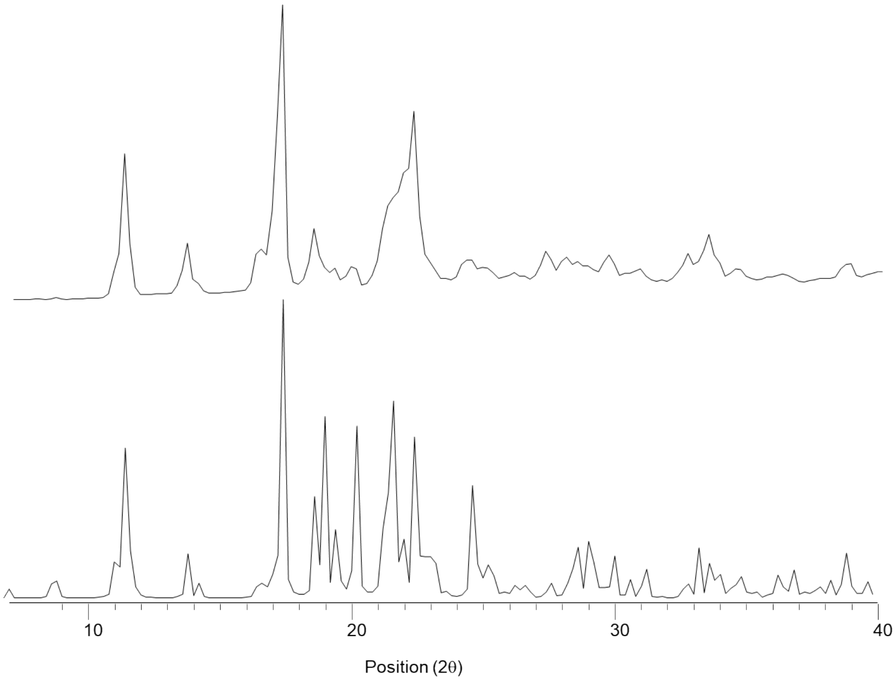

Figure S5. XRPD pattern of the isolated crystals of **1** (top) and theoretical pattern calculated from the single crystal data (bottom).

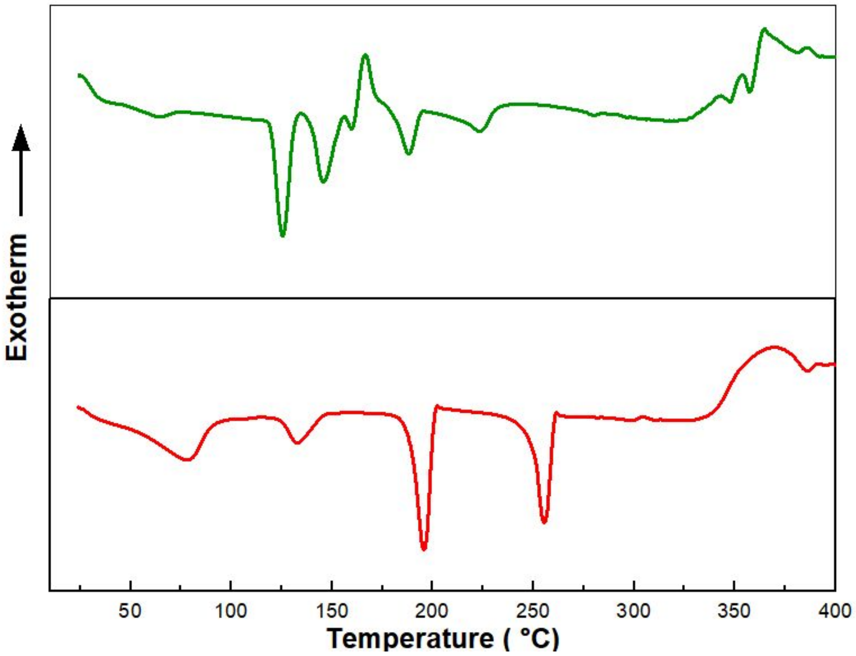

Figure S6. DSC plots of **1** (top) and of commercial aspartame (bottom).

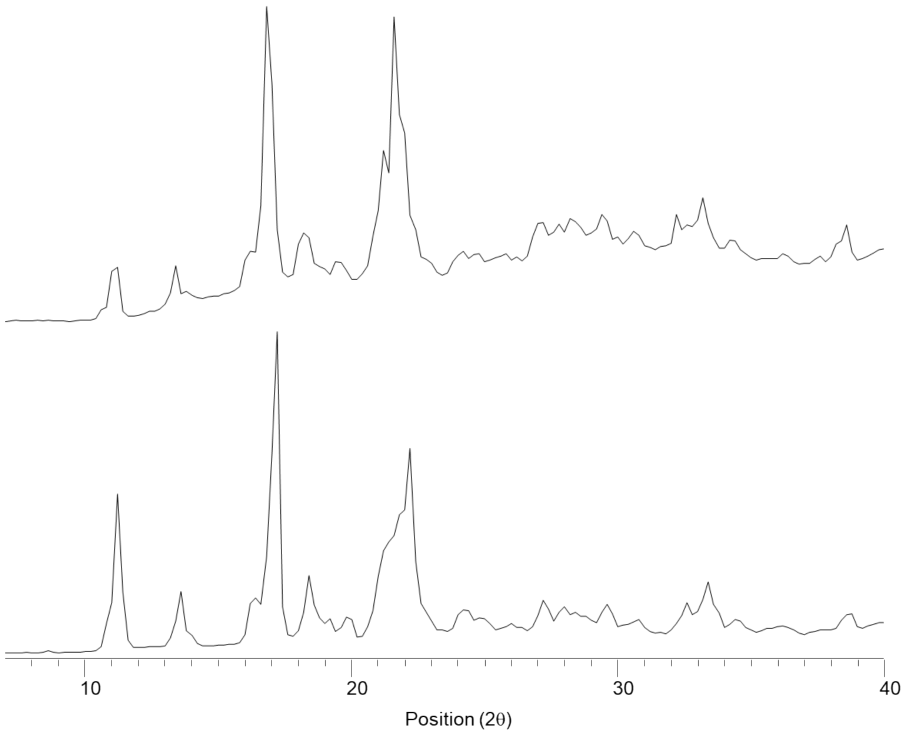

Figure S7. XRPD pattern of the isolated crystals of **1** directly after isolation (bottom) and after storage at 56 % RH for two weeks (top).

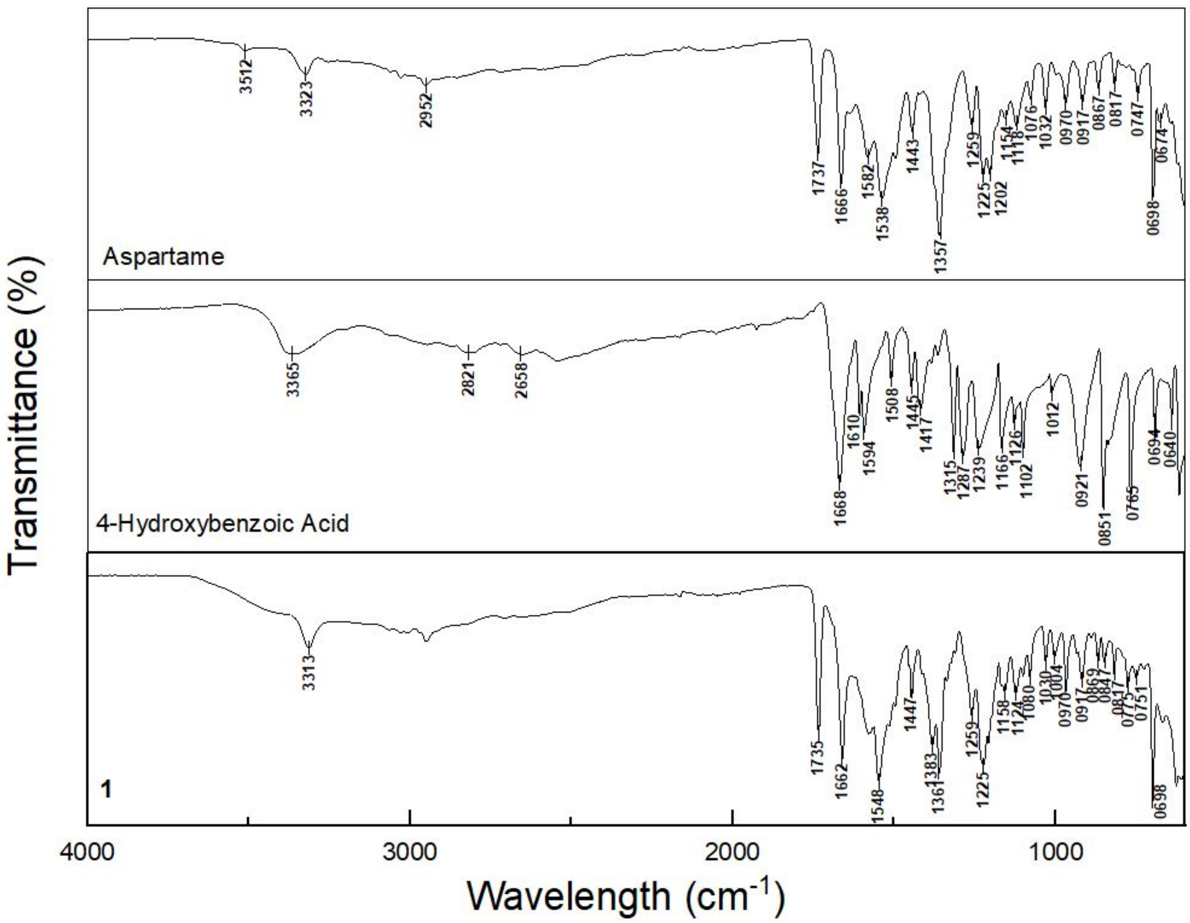

Figure S8. IR spectra of aspartame, 4-hydroxybenzoic acid and **1**.
